# Evaluation of a probiotic blend on psychosocial health and biomarkers of inflammatory, immune and stress response in adults with subthreshold depression: a double-blind, randomised, placebo-controlled trial

**DOI:** 10.1017/S0007114524001703

**Published:** 2026-03-14

**Authors:** George Moschonis, Katerina Sarapis, Stephanie Resciniti, Renate Hall, Kanny Yim, Matilda Tonkovic, Clare Fitzgerald, Fay Anixiadis, Antony Vinh, Quynh Nhu Dinh, Rachael A. Cronin, Matthew W. Hale, Bradley J. Wright, Marco Pane, Caroline J. Tuck, Jessica R. Biesiekierski

**Affiliations:** 1 Discipline of Food, Nutrition and Dietetics, Department of Sport, Exercise and Nutrition Sciences, School Allied Health, Human Services and Sport, La Trobe University, Melbourne, VIC 3086, Australia; 2 La Trobe Institute for Sustainable Agriculture & Food (LISAF), La Trobe University, VIC 3086, Australia; 3 Centre for Cardiovascular Biology and Disease Research (CCBDR), La Trobe Institute of Medical Science (LIMS), La Trobe University, Melbourne, VIC 3086, Australia; 4 Department of Microbiology, Anatomy, Physiology & Pharmacology, School of Agriculture, Biomedicine and Environment, La Trobe University, Melbourne, VIC 3086, Australia; 5 Department of Psychology, Counselling and Therapy, La Trobe University, Albury Wodonga, VIC 3690, Australia; 6 Department of Psychology, Counselling and Therapy, La Trobe University, Melbourne, VIC 3086, Australia; 7 Probiotical Research srl, Novara, 28100, Italy; 8 Department of Nursing and Allied Health, Swinburne University, Melbourne, VIC 3122, Australia; 9 Department of Nutrition, Dietetics and Food, Monash University, Melbourne, VIC 3168, Australia

**Keywords:** Depression, Subthreshold depression, Anxiety, Probiotics, Lactobacillus

## Abstract

This study examined the efficacy of a probiotic in reducing depressive symptom severity in people with subthreshold depression. In a double-blind, randomised, placebo-controlled trial, a probiotic (1 × 10^9 live cells per strain: *Limosilactobacillus fermentum* LF16 (DSM26956), *Lacticaseibacillus rhamnosus* LR06 (DSM21981), *Lactiplantibacillus plantarum* LP01 (LMG P-21021) and *Bifidobacterium longum* 04 (DSM23233)) or placebo was taken daily for 12 weeks. Data were collected at baseline, 6 and 12 weeks including psychological symptom severity (Beck Depression Inventory, BDI; Patient Health Questionnaire, PHQ; Hospital Anxiety Depression Scale, HADS; and Depression Anxiety and Stress Scale, DASS). Biomarkers of glycaemia, inflammation (high-sensitivity C-reactive protein, hs-CRP), antioxidant status (total glutathione (GSH)) and stress (cortisol awakening response, CAR) were also measured. Thirty-nine participants (nineteen probiotic; twenty placebo) were enrolled. There were no significant between-group differences in the examined psychological symptom severity scores, despite certain significant within-group changes observed in both groups from baseline to 6 and/or 12 weeks of follow-up. Regarding biomarkers, the probiotic group showed reduced hs-CRP (–1520; 95 % CI –273·7, −2766·2 ng/dl) and CAR (–0·28; 95 % CI −0·05, −0·51 μg/dl) at 12 weeks, but increased total GSH (3·9; 95 % CI 0·1, 7·5 ng/dl) at 6 weeks, compared with the placebo. The current study reported favourable decreases in depressive symptoms in both groups. Although the within-group changes observed in the probiotic group were supported by favourable inflammatory, antioxidant status and stress biomarker changes compared with the placebo, further research is required to shed more light on the role of gut microbiota modulation on emotional regulation.

Depression has emerged as the leading cause of global disability, with an estimated 322 million individuals affected^(1)^. However, a substantial portion of those impacted do not meet the diagnostic criteria for major depressive disorder (MDD), falling into the category referred to as subthreshold depression (SD)^(2–4)^. The proposed definition of SD requires individuals experiencing at least two core depressive symptoms lasting for 2 weeks or more, one symptom of depressed mood, and is ideally based on additional patient- or caregiver-based rated scales^(5)^. However, there is considerable heterogeneity underlying the concept of SD. These cases of mild depressive conditions (also referred to as subclinical or subsyndromal depression) are associated with increased functional and social impairment^(2)^, increased utilisation of healthcare^(6)^ and an elevated risk of MDD^(7,8)^. The lifetime prevalence of SD ranges from 2 % to 21 % worldwide^(9)^, with SD suggested to be twice as common as MDD^(4,6)^, while about 8 % of individuals with SD go on to develop MDD each year^(10)^. In this regard, early identification of SD and timely and effective intervention is key to MDD prevention.

Current international best practice management guidelines for SD recommend active monitoring as an initial approach, with up to 70 % of cases resolving spontaneously^(11)^. If symptoms persist, individually facilitated self-help programmes, structured physical activity or peer support programmes are recommended^(12)^. If no improvement occurs, a step-up approach is taken with interventions encompassing both pharmacological and psychological treatments. While pharmacological treatments and cognitive therapies have proven effective for moderate to severe MDD, their limitations of cost, accessibility^(13–15)^ and stigma^(16)^ make them less suitable for SD. Further, systematic reviews have shown that antidepressants do not outperform placebos in treating minor depression^(17)^ and antidepressants have been linked to adverse reactions^(18)^. Even those who do seek treatment may not achieve full remission or may still have subclinical depression despite an initial response^(19)^. Given the wide-reaching impact of depression and the challenges associated with treatment, there is a pressing need for novel, population-based, accessible and evidence-based treatment and prevention strategies for SD.

The pathophysiology of depression is complex, encompassing interactions with the central nervous system and with persistent low-grade inflammation. A potential causal relationship has been proposed between the gut microbiota and depression^(20)^, as the gut microbiota plays a role in the development and functioning of the immune system and the brain through the microbiota–gut–brain axis. This axis is crucial for maintaining homoeostasis and modulating neuropsychological functions of the central nervous system. Consequently, dysregulation of the microbiota–gut–brain axis has been implicated in the aetiology of various metabolic and mental health disorders, including depression^(21)^.

There has been growing interest in non-pharmacological approaches for preventing or treating depressive symptoms. In this regard, recent research has focused on probiotics, which as per their definition by the WHO represent ‘live microorganisms that when administered in adequate amounts will confer a health benefit on the host’ as an adjunctive therapy for depression^(22)^. This emerging field, often termed ‘psychobiotics’^(23)^ explores the effects of beneficial bacteria (probiotics) or substances that support such bacteria (e.g. prebiotics) on the brain. These substances exert their influence through microbiome-mediated psychological effects via the gut–brain axis, with both animal and human studies indicating potential benefits of probiotics on central nervous system function^(24)^. Probiotics may modulate brain activity, possibly due to the production of various signalling molecules, involved in neuroactive, neuroendocrine and immunomodulatory pathways^(25)^. Evidence from randomised controlled trials and meta-analyses supports the efficacy of probiotics in reducing clinical depression and depressive-like symptoms in MDD patients, particularly when used as adjuncts to conventional antidepressants or other clinical management strategies^(26–31)^. Proof-of-concept data suggest that a probiotic multi-species formulation may be effective in alleviating depressive symptoms, as a 6-week double-blind, placebo-controlled study involving thirty-eight healthy volunteers reported improvements in mood, depressive mood state, anger, fatigue and sleep quality^(32)^. Furthermore, in another 6-week randomised, double-blind, placebo-controlled trial, supplementation of another multi-species probiotic resulted in improvements in several questionnaire-derived scores evaluating mood, anxiety and depression when administered to seventy healthy men and women^(33)^.

Despite the promising evidence that probiotics are effective in reducing depressive symptoms in human studies, there are limitations including lack of consistent and reproducible evidence of mechanism, and support in wider clinical populations, including people experiencing depressive symptoms across all severity categories. Given that SD represents a significant but reversible preclinical condition, early clinical intervention and prevention is essential. Therefore, this study aimed to evaluate a multi-species probiotic’s efficacy in reducing depressive symptom severity in patients diagnosed with SD in comparison with a placebo. Secondary aims included assessing the probiotic’s impact on quality of life and psychosocial indicators (e.g. anxiety, stress and mood) and examining its effects on inflammatory, antioxidant and stress response biomarkers. The study hypothesised that the probiotic group would experience a reduction in depressive symptom severity compared with placebo, as well as improved levels of inflammatory and immune markers.

## Materials and methods

The study was conducted in compliance with the NHMRC National Statement on Ethical Conduct in Human Research (2007), the Note for Guidance on Good Clinical Practice (CPMP/ICH-135/95) and the CONSORT statement. All procedures involving human subjects were approved by the Human Research Ethics Committee of La Trobe University (HEC21032), and written informed consent was obtained from all volunteers. The trial protocol was registered with the Australia New Zealand Clinical Trials Registry (ACTRN12621000675820).

### Study participants

All participants were recruited in Melbourne, Australia, between May and September 2022, via social media, flyers, word-of-mouth and a research recruitment company (Trialfacts, Melbourne, Australia). The Structured Clinical Interview for DSM-5 (SCID-5)^(34)^ was used during the screening phase (by phone or video call) with the study psychologist to exclude any specified depressive disorder diagnosis in the DSM-5 (Diagnostic and Statistical Manual of Mental Disorders) and assess the symptoms of SD (i.e. participants had to meet one of the two core symptoms for depressions, such as low or depressed mood, markedly diminished interest or pleasure in all or almost all activities most of the day, nearly every day, during a time period of at least 2 weeks before diagnosis), as well as one other symptom (i.e. low energy or fatigue, feelings of guilt, low self-esteem, insomnia, etc.). Eligible participants were required to be within the age range of 18 and 65 years, with a BMI ≥ 18·5 kg/m^2^, not been taking antidepressants or other medications acting on the central nervous system for at least 6 weeks prior to the initiation of the intervention and have access to the Internet and be able to understand English for completion of study questionnaires. Exclusion criteria included patients with substance use or organic mental disorders and/or patients with neurological conditions affecting the brain or other central functions, any suicidal ideation/ risk of harm; patients with organic co-morbidities affecting the gastrointestinal tract, or autoimmune or chronic inflammatory conditions; individuals taking nutritional supplements (i.e. probiotics, vitamins, minerals and antioxidants) or antibiotics for at least 6 weeks prior to the study intervention commencement; and pregnant or breast-feeding women.

### Study design and intervention

The study was a double-blind, randomised, placebo-controlled, clinical trial conducted at the ‘Food, Nutrition and Dietetics’ Research Laboratory at La Trobe University, Melbourne. Enrolled participants were randomly assigned in a 1:1 ratio, to one of the two treatment groups, that is, probiotic food supplement or placebo, following a block randomisation process (*n* 17 blocks, ‘10 blocks of 2’ and ‘7 blocks of 4’), generated via the use of an online random sequence generation tool (http://randomization.com). An independent senior researcher not otherwise involved in the study generated and stored the sequence. The research team and participants were blinded until data collection and statistical analysis were complete.

Study participants were requested to ingest the probiotic or placebo supplement (one capsule) daily for 3- months (12 weeks). The product is a mix of selected probiotic bacteria, with one capsule weighing 355 mg and containing active ingredients comprised of a mixture of lyophilised probiotic strains of the following genus: *Limosilactobacillus fermentum* LF16 (DSM26956), *Lacticaseibacillus rhamnosus* LR06 (DSM21981), *Lactiplantibacillus plantarum* LP01 (LMG P-21021) and *Bifidobacterium longum* 04 (DSM23233), all with strain-specific activity, being extensively used for food supplement formulations. The live cell count for each strain is 1 × 10^9. This probiotic mixture has previously shown positive effects on mood state including a reduction in depressive mood in healthy volunteers^(32)^. The probiotic is commercially available as Bifizen^TM^ (Europe and USA) and as Biome Lift™ (Australia). The placebo contained the same non-active ingredients (Glyceryl palmitostearate (E471), Silicon dioxide, Maltodextrin, Hypromellose, Titanium dioxide) as the probiotic capsules, except for the active ingredients (i.e. the bacteria).

Both probiotic and placebo were shelf-stable and were administered to participants at baseline, packaged in blister packs that contained the same number of capsules provided by the study sponsor (Biome Australia Trading Pty Ltd). To ensure blinding to the supplement type, the probiotic and placebo capsules had identical appearance (i.e. size, colour and texture), while each blister pack was assigned a different code number that was concealed from study participants and research team members. The code was retained by the researcher who generated the sequence and only disclosed after completion of the statistical analyses. Reminders to aid compliance of probiotic or placebo consumption included daily prompts (via a push notification by the Medisafe App) and the blister pack being clearly labelled with the days of the week. To assess the level of compliance, participants were instructed to record the number of any unconsumed supplements (capsules) at the end of the intervention period. The probiotic product was analysed (Biolab SRL) via flow cytometry (ISO 19344, 2015: IDF 232:2015) upon batch release which resulted in a total viable cell count of > 4 × 10^9^ per capsule and plate count method as colony-forming units (Internal Method 014–06).

### Measurements

**Fig. 1.**
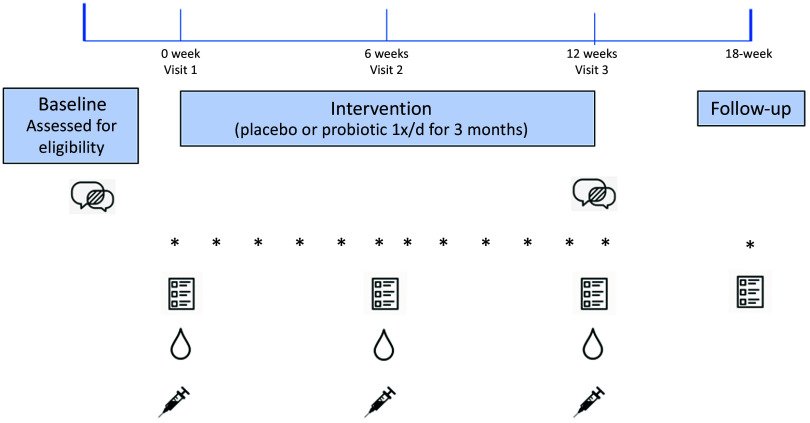
Overview of study protocol. Clinical psychologist interviews were conducted pre- and post-intervention. Asterisks indicate weekly primary end point questionnaire. All other questionnaires were completed pre-, mid- and post-intervention, with an optional follow-up time point. Saliva and blood samples were collected pre-, mid- and post-intervention.

Data were collected at three study visits (baseline, mid-intervention at 6 weeks and immediately post-intervention at 12 weeks). Participants were also invited to complete all online questionnaires at 6 weeks post-supplementation for a ‘Follow-up’ of data collection (this was optional). The study protocol and measurements are summarised in Fig. 1.

### Socio-demographics

Socio-demographic information was collected from participants during the screening phase and baseline visit. The socio-demographic data collected included level of education, employment status, ethnicity and country of birth. To evaluate individuals’ health status (e.g. weight, height), lifestyle habits (e.g. smoking) and other relevant medical information (e.g. medical history and use of medication and supplements) were also recorded.

### Dietary intake

A handwritten 3-d food diary was used to collect information on the dietary intake of study participants during two weekdays and one weekend day (preferably non-consecutive) at baseline, 6 weeks and 12 weeks. Participants were instructed to record details on their intake of food and beverages, including information on the quantity, type/brand and cooking methods of the consumed items. The completed food diaries were returned and checked by a research team member for potential incorrect or missing entries during each scheduled study visit. FoodWorks®10 software (Xyris Software Pty Ltd) was used for assessing dietary intake and extracting data on energy, micro- and macronutrients as well as the consumption of food groups and individual food items.

### Physical activity

Physical activity (PA) was assessed using the Active Australia Survey (AAS) questionnaire^(35)^, a tool that has been validated in the Australian population. This questionnaire is designed to assess participation in a range of leisure-time physical activities of light, moderate and vigorous intensity. The questionnaire consists of eight questions, which assess the number of sessions and total weekly time spent in activities of different intensity. Participants were required to complete and submit the AAS questionnaire during the week preceding the baseline and post-intervention study visits. The amount of time (in minutes per d) that study participants were engaged in physical activity of different intensity was calculated.

### Anthropometric measurements

Anthropometric measurements were taken at all three study visits (baseline, 6 weeks and 12 weeks). Body weight and standing height were measured with study participants in light clothing and barefoot, using a column scale and stadiometer (SECA 703) to the closest 0·1 kg and to the nearest 0·1 cm, respectively. BMI was calculated using Quetelet’s equation (weight (kg)/height (m)^2^.

### Questionnaires

Questionnaires that measure severity of depressive symptoms, anxiety, stress and quality of life were filled in by study participants at baseline, 6 weeks and 12 weeks. These self-reported questionnaire data were collected and managed using REDCap (Research Electronic Data Capture) tools hosted at La Trobe University^(36)^. Specifically, the *Beck Depression Inventory (BDI-II)* ^(37)^ was used to measure the presence and degree of existing depression in study participants with particular reference to the cognitive and the somatic-affective areas. It consists of twenty-one items, each of which is rated on a four-point Likert scale, ranging from 0 to 3. Total scores range from 0 to 63, with high scores indicating severe levels of depression.

A brief online depression tool (*Patient Health Questionnaire (PHQ9))* ^(38)^ that specifically measures depression was also completed weekly during the 3-month supplementation phase. The PHQ-9 is a scale that asks respondents to rate their experience of nine symptoms from 0 to 3; a total PHQ-9 score of 10 or more indicates moderate to severe symptoms, while scores of 5–9 indicate mild symptoms.

Anxiety and depression symptoms severity were assessed with the use of *the Hospital Anxiety and Depression Scale (HADS)* ^(39)^, which is a validated fourteen-item questionnaire. Each question was scored between 0 and 3 (no impairment *v*. severe impairment, respectively), with a maximum score of 21 for anxiety or depression.

Given the HADS does not capture all aspects of depression, multiple tools were used. Participants’ negative emotional states of depression, anxiety and stress were measured with the *Depression, Anxiety and Stress Scale (DASS-21)* ^(40)^. The DASS-21 is a set of three seven-item self-report scales divided into subscales with similar content. Each item comprises a statement and four short response options to reflect severity, scored from 0 to 3. Scores for depression, anxiety and stress are calculated by summing the scores for the relevant items, with higher ratings indicating more severe symptoms. Stress perception was also measured by the *Perceived Stress Scale (PSS)* ^(41)^, a fourteen-item self-administered questionnaire with questions worded both positively and negatively and rated on a five-point Likert scale, ranging from 0 to 4.

The *Assessment of Quality of Life (AQoL-8)* ^(42)^ was used to assess parameters of well-being, with a focus on psychosocial elements of health. This self-report thirty-five-item questionnaire uses eight separately scored dimensions (consisting of AQoL-6D and two additional dimensions) to give a global score representing range of good health. Item responses are assigned a psychometric value, with higher scores indicating ‘poor quality of life’.

A research team member reviewed the completed questionnaires within 1 week of completion. If a participant had a score that raised any concern (i.e. BDI scores that range from 29 to 63 indicative of severe depression and/or scoring high on questions related to risk of self-harm), this was flagged to the study psychologist and contact was made with the participant and referred to their general practitioner if and whenever appropriate.

### Biological samples and laboratory analyses

#### Blood collection

Venous blood was collected from participants (in the morning following a 10–12 h overnight fast) by a trained phlebotomist at La Trobe University and at three time points (baseline, 6 weeks and 12 weeks). Collected venous blood was centrifuged (Hettic Rotina 420r) at 2739 rcf for 10 min at 4°C, and the extracted plasma and/or serum was apportioned into aliquots of 500 μl each and stored at −80°C until analysis.

Serum insulin was measured by ELISA (KAQ1251, Thermo Fisher Scientific) (Analytical sensitivity: 0·17 μIU/ml; Assay range: 5·1–250 μIU/m). Plasma glucose was measured by ELISA (EIAGLUC, Thermo Fisher Scientific) (Analytical sensitivity: 0·413 mg/dl; Assay range: 0·5–32 mg/dl). Homoeostatic Model Assessment for Insulin Resistance (HOMA-IR) was calculated according to the formula: Fasting insulin (microU/l) × Fasting glucose (mmol/l)/22·5. Serum high-sensitivity C-reactive protein (hs-CRP) was measured by ELISA (BMS288INST, Thermo Fisher Scientific) (Analytical sensitivity: 3·0 pg/ml; Assay range: 78–5000 pg/ml). All hs-CRP values were also screened for the identification of any cases of acute infections. However, there was no case identified with serum hs-CRP concentration higher than 10 mg/l, which is the threshold for the identification of acute infections. Plasma total glutathione (GSH) was measured by ELISA (Ab239709, Abcam) (Assay range: 5–500 ng/ml). All ELISA protocols were followed as per manufacturer’s instructions. The absorbance was measured at 415 nm for total GSH, 450 nm for insulin and CRP, and 560 nm for hs-CRP using a Clariostar Plus Microplate reader (BMG; Labtech). The concentration of protein was estimated from the standard curve using GraphPad Prism 9.

#### Saliva sample collection

The Salivette® Oral Swabs (Sarstedt) were used to collect saliva samples to measure cortisol awakening response (CAR) levels. Participants were provided with written instructions on sample storage (–20°C) and collection that were largely consistent with expert consensus guidelines^(43)^. Participants (all daytime employees) were asked to provide samples upon awakening between 06.30 and 07.30 (S1) and 30 min after waking (S2) on the day before each study visit. The CAR was calculated by subtracting S2 to S1. Samples were frozen until analysis, which was conducted with a high-sensitivity salivary cortisol enzyme immunoassay kit (No. 1–3002, Salimetrics, LLC) according to manufacturer’s instructions^(44)^.

#### Adverse event reporting

Adverse events information was collected from study participants during follow-up phone calls and was recorded in the withdrawal form. The frequency of each adverse event was reported based on severity ratings such as ‘mild’, ‘moderate’ or ‘severe’.

#### Sample size

Sample size was determined based on the differences in the mean reductions of BDI-II scores between the mean changes from pre- to post intervention^(45)^. Based on this calculation, a sample size of forty participants (twenty in each group) was determined to provide at least 80 % power with two-sided type I error of 0·05 in order to detect a mean difference of 4·2 (minimal important change difference) between the probiotic and the placebo group. As we predicted a probable loss to follow-up of 20 %, we considered a total sample size of 48 at the recruitment stage.

### Statistical analysis

Statistical analyses were performed using the SPSS statistical analysis software for Windows (version 25.0). Five multiple imputations were conducted to estimate missing values, thus allowing intention-to-treat analyses with the maximum possible sample size. All continuous variables were checked for the normality of their distribution, with normally distributed variables presented as mean and standard deviation. General linear models were used to examine the between-group differences (treatment effect, i.e. probiotic *v*. placebo) of mean values at each time point of measurement, the within-group changes (time effect) from baseline to follow-up time points in each treatment group, and the differences in the changes from baseline to follow-up between the two treatment groups (treatment × time interaction effect). Bonferroni corrections were applied in *post hoc* multiple comparisons to account for type I error, while all analyses were adjusted for those variables that were found to differ significantly between the two treatment groups. All reported *P*-values were two-tailed, while the level of statistical significance was set at *P* < 0·05.

## Results

### Participant characteristics

**Fig. 2.**
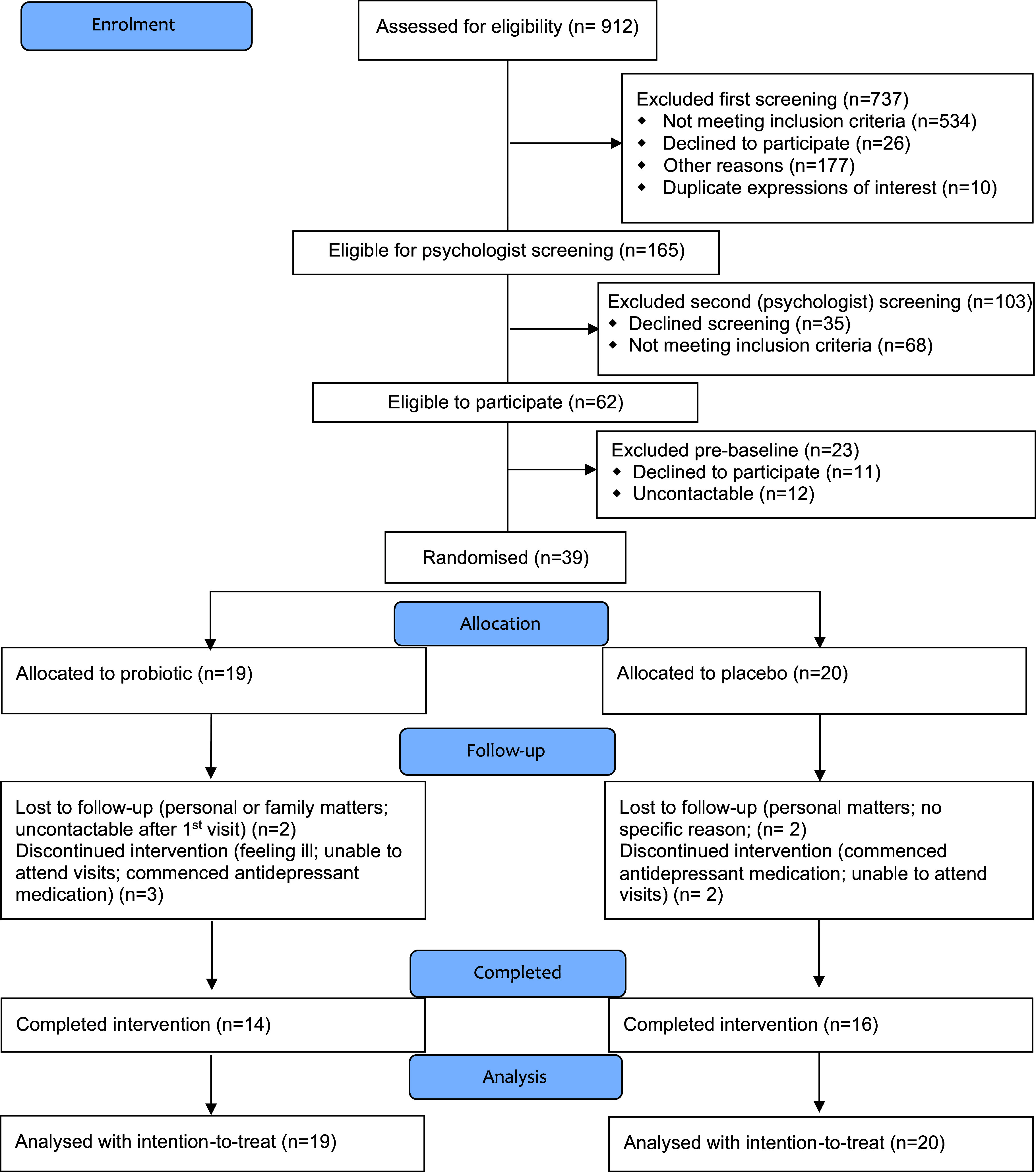
Study participant flow diagram (CONSORT).

From the 912 volunteers who expressed interest in participating to the study, 737 were excluded in the initial screening phase due to either not meeting the eligibility criteria or due to other reasons (e.g. declined to participate, duplicate expressions of interest, as indicated in Fig. 2). This allowed 165 volunteers to be referred to the second screening phase with the study psychologist. Through the second screening, sixty-two volunteers were found to be eligible to participate in the study (103 did not meet the inclusion criteria or declined screening). However, as twenty-three out of the sixty-two eligible volunteers were either uncontactable or unwilling to participate, a final number of thirty-nine eligible volunteers agreed to participate and were randomly allocated to one of the two treatment groups (*n* 19, probiotic group; *n* 20, placebo group). Due to losses to follow-up or discontinuation of the intervention, at the end of the 12-week intervention period, complete data were collected from thirty study participants (i.e. *n* 14, probiotic group; *n* 16, placebo group) (Fig. 2). Data from nineteen participants were collected at the optional follow-up time point of 6 weeks post-intervention (see Supplementary material).

### Supplementation compliance

Compliance data were captured from eighteen participants, assessed by the number of remaining capsules at the end of the intervention (12 weeks). Specifically, compliance in the probiotic group (*n* 7) was 100 % (with 0 capsules left unconsumed). In the placebo group (*n* 11), the number of unconsumed capsules ranged from 0 to 3 (*n* 8 and *n* 3 participants, respectively).

### Adverse event reporting

Adverse events were self-reported throughout the study. Only one participant from the probiotic group reported feeling ill and experiencing mild to moderate symptoms of reflux and nausea, and who withdrew within the first week of participation. During the concurrent questionnaire checks completed throughout the study, four participants were identified as scoring high (2 or above on questions related to self-harm on the BDI) and referred to the study psychologist. However, none of these were subsequently referred to their general practitioner or psychologist.

### Socio-demographics, anthropometrics and dietary intake data

Table 1 summarises the descriptive characteristics of study participants, in terms of socio-demographics and anthropometrics, in the total sample (*n* 39) and by treatment group. There was no difference observed between the two treatment groups in almost all descriptive characteristics, thus indicating homogeneity at baseline. The only exemption was participants’ education level, with higher percentage of participants allocated to the probiotic group having a postgraduate degree compared with those allocated to the placebo group (36·9 % *v*. 15·0 %, *P* < 0·05).

**Table 1.** Baseline differences in demographic and anthropometric indices between treatment groups (Mean values and standard deviations; numbers and percentages)

	Placebo (*n* 20)		Probiotic (*n* 19)		*P* ^*^
	Mean	sd	Mean	sd	
Age (years)	41·8	11·4	42·9	15·8	0·804
Weight (kg)	83·4	21·3	76·1	18·5	0·257
Height (cm)	170·1	8·3	166·3	10·1	0·207
BMI (kg/m^2^)	28·8	6·6	27·6	6·4	0·568
	*n*	%	*n*	%	
BMI categories					0·413
Normal weight	6	30·0	9	47·4	
Overweight	7	35·0	3	15·8	
Obese	7	35·0	7	36·8	
Sex					0·365
Males	8	40·0	5	26·3	
Females	12	60·0	14	73·7	
Educational level					**0·024**
Apprentice/Trade	2	10·0	0	0·0	
Secondary school	0	0·0	4	21·1	
Certificate/Diploma	7	35·0	2	10·5	
Bachelor’s degree	8	40·0	6	31·6	
Postgraduate (Master’s/PhD)	**3**	**15·0** ^*^	**7**	**36·9** ^*^	
Employment status					0·443
Unemployed	3	15·0	1	5·3	
Casual employment	4	20·0	4	21·1	
Part-time employment	5	25·0	2	10·5	
Full-time employment	8	40·0	11	57·9	
Retired	0	0·0	1	5·3	
Place of birth					0·763
Australian/New Zealander	16	80·0	15	78·9	
European	1	5·0	2	10·5	
Asian	3	15·0	2	10·5	
Region of residence					0·064
Urban	20	100·0	16	84·2	
Rural	0	0·0	3	15·8	

Data are presented as mean and standard deviation or as frequencies (*n*) and percentages (%).

* Derived from Student’s *t* test for continuous variables and from *χ* ^2^ test for categorical variables. Values in bold indicate statistical significance (*P* < 0·05) in the comparisons between treatment arms at baseline.

Table 2 illustrates the changes observed in body weight and BMI from baseline to the follow-up time points, the between-group differences (i.e. at 6 and 12 weeks) and the treatment × time interaction effects. There were no within-group changes, between-group differences or treatment × time interaction effects observed in the examined anthropometric indices. Considering that changes (especially increases) in weight status/body size can be associated with changes in anxiety and stress levels, the non-existence of between-group differences or within-group changes in anthropometric indices indicates that there is no confounding effect of weight status on the study outcomes.

**Table 2.** Changes in anthropometric measurements of people diagnosed with subthreshold depression receiving either a probiotic food supplement (*n* 19) or a placebo (*n* 20), from baseline to 6 and 12 weeks of intervention (Mean values and standard deviations; mean values and 95 % confidence intervals)

	Baseline		6-week follow-up		6-week change		12-week follow-up		12-week change	
	Mean	sd	Mean	sd	Mean	95 % CI	Mean	sd	Mean	95 % CI
Body weight (kg)										
Placebo	83·4	21·3	84·7	21·5	1·2	–12·0, 14·5	85·0	19·9	1·6	–11·6, 14·8
Probiotic	76·1	18·5	76·5	17·7	0·4	–11·4, 12·2	77·0	18·3	0·9	–10·8, 12·8
* P* ^†^	0·257		0·202		0·660^*^		0·200		0·768^*^	
BMI (kg/m^2^)										
Placebo	28·8	6·6	29·2	6·9	0·5	–3·6, 4·5	29·2	5·7	0·5	–3·6, 4·6
Probiotic	27·6	6·4	27·7	6·1	0·2	–3·9, 4·3	28·0	6·4	0·4	–3·7, 4·5
* P* ^†^	0·568		0·477		0·687^*^		0·513		0·913^*^	

* Treatment × Time interaction effect.

† Between-groups’ differences in mean values at baseline, 6 and 12 weeks, as well as in 6- and 12-week changes from baseline (treatment effect).

The within-group changes observed in dietary energy and macronutrients’ intake from baseline to follow-up time points as well as the between-group differences and the treatment × time interaction effects are summarised in Table 3. Overall, there were no within-group changes, between-group differences or treatment × time interaction effects observed for the vast majority of dietary intake indices. However, a decrease was observed for energy intake from baseline to the 6-week (–1683·4, 95 % CI −3080·0, −286·8 kJ/d) and 12-week follow-up (–1467·5, 96 % CI −2864·01, −70·9 kJ/d) only in the placebo group. Similarly, protein intake was found to decrease from baseline to the 12-week follow-up, but only in the probiotic group (–2·5, 95 % CI –4·8, −0·7). Finally, dietary fibre intake was found to be higher in the probiotic compared with the placebo group (2·8 (sd 1·4) *v*. 2·0 (sd 0·8), *P* = 0·045) at the 6-week follow-up but not at 12-week follow-up.

**Table 3.** Changes in dietary intake of people diagnosed with subthreshold depression receiving either a probiotic food supplement (*n* 19) or a placebo (*n* 20), from baseline to 6 and 12 weeks of intervention (Mean values and standard deviations; mean values and 95 % confidence intervals)

	Baseline		6-week follow-up		6-week change		12-week follow-up		12-week change	
	Mean	sd	Mean	sd	Mean	95 % CI	Mean	sd	Mean	95 % CI
Energy intake (kJ/d)										
Placebo	8942·8	2537·5	7259·3	1901·8	**–1683·4**	**–3080·0, −286·8**	7475·2	2130·1	**–1467·5**	**–2864·01, −70·9**
Probiotic	7733·4	2081·5	7613·6	1189·5	–119·8	–1221·1, 981·4	7645·5	1688·6	–87·9	–1189·2, 1013·4
* P* ^†^	0·113		0·493		**0·050** ^*^		0·784		0·113^*^	
Protein intake (% kJ)										
Placebo	19·7	5·2	19·7	4·7	0·01	–3·0, 3·0	18·9	4·4	–0·7	–3·8, 2·3
Probiotic	19·3	4·1	18·5	3·0	–0·8	–3·1, 1·5	16·8	3·4	**–2·5**	**–4·8, −0·7**
* P* ^†^	0·806		0·361		0·590^*^		0·109		0·419^*^	
Carbohydrates intake (% kJ)										
Placebo	39·3	10·5	38·6	7·2	–0·7	–6·2, 4·8	37·7	8·2	–1·5	–7·1, 4·0
Probiotic	37·7	5·5	39·1	7·3	1·4	–2·8, 5·6	41·1	6·4	3·4	–0·7, 7·6
* P* ^†^	0·555		0·830		0·503^*^		0·160		0·111^*^	
Total fibre intake (% kJ)										
Placebo	2·1	0·6	**2·0**	**0·8**	–0·1	–0·6, 0·3	2·1	0·9	–0·1	–0·6, 0·4
Probiotic	2·3	0·9	**2·8**	**1·4**	0·4	–0·3, 1·2	2·7	1·2	0·3	–0·5, 1·1
* P* ^†^	0·446		**0·045**		0·151^*^		0·079		0·237^*^	
Total fat intake (% kJ)										
Placebo	34·9	6·6	34·4	5·9	–0·5	–5·1, 4·2	35·8	9·1	0·9	–3·7, 5·6
Probiotic	35·7	6·2	35·1	5·7	–0·6	–4·3, 3·1	36·7	5·2	1·0	–2·7, 4·8
* P* ^†^	0·689		0·731		0·931^*^		0·705		0·976^*^	
Saturated fat intake (% kJ)										
Placebo	13·1	3·5	12·7	2·8	–0·4	–5·1, 4·2	13·1	2·5	–0·01	–1·9, 1·9
Probiotic	13·4	3·9	12·4	2·7	–1·0	–3·2, 1·2	14·0	3·4	0·6	–1·6, 2·8
* P* ^†^	0·797		0·742		0·588^*^		0·327		0·623^*^	
Monounsaturated fat intake (% kJ)										
Placebo	13·3	3·8	13·4	3·8	0·2	–2·9, 3·2	14·1	6·4	0·8	–2·2, 3·9
Probiotic	13·8	3·8	13·8	3·7	–0·01	–2·3, 2·3	14·0	3·0	0·2	–2·1, 2·5
* P* ^†^	0·687		0·793		0·906^*^		0·929		0·667^*^	
Polyunsaturated fat intake (% kJ)										
Placebo	5·6	1·7	5·3	1·5	–0·3	–1·6, 0·9	5·6	2·7	–0·05	–1·3, 1·2
Probiotic	5·4	2·0	5·4	2·0	–0·01	–1·3, 1·3	5·6	2·1	0·2	–1·1, 1·5
* P* ^†^	0·715		0·851		0·755^*^		0·956		0·755^*^	
Alcohol intake (% kJ)										
Placebo	3·1	3·0	4·9	6·8	1·8	–1·4, 5·1	3·9	4·7	0·8	–2·4, 4·0
Probiotic	4·1	5·3	3·9	3·4	–0·2	–2·8, 2·4	4·1	3·0	–0·02	–2·6, 2·6
* P* ^†^	0·445		0·577		0·239^*^		0·843		0·633^*^	

Values in bold indicate statistical significance (*P* < 0·05) of within-group changes from baseline to 6 or 12 weeks of intervention.

* Treatment × Time interaction effect.

† Between-groups’ differences in mean values at baseline, 6 and 12 weeks, as well as in 6- and 12-week changes from baseline (treatment effect).

### Mood-related outcomes

**Fig. 3.**
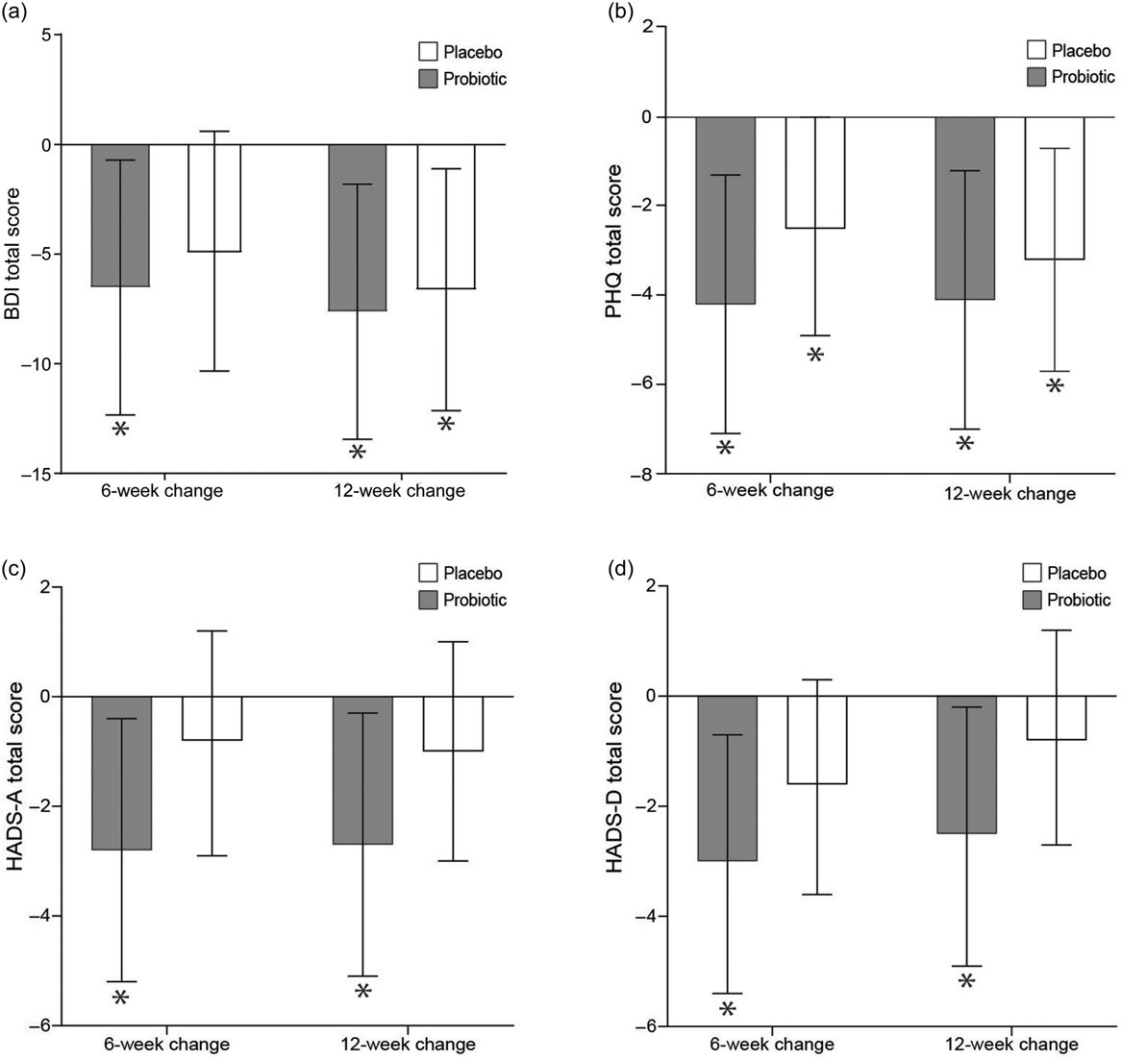
Mean changes (95 % CI) of depression and anxiety total scores in people diagnosed with subthreshold depression receiving either a probiotic food supplement (*n* 19) or a placebo (*n* 20), from baseline to 6 and 12 weeks of intervention. These changes refer to (a) BDI, Beck Depression Inventory scores, (b) PHQ, Patient Health Questionnaire scores, (c) HADS-A, Hospital Anxiety and Depression Scale-A scores and (d) HADS-D, Hospital Anxiety and Depression Scale-D scores. *P < 0·05, significant within-group change from baseline to follow-up. No significant treatment × time interaction effect was observed at 6 or 12 weeks.

**Fig. 4.**
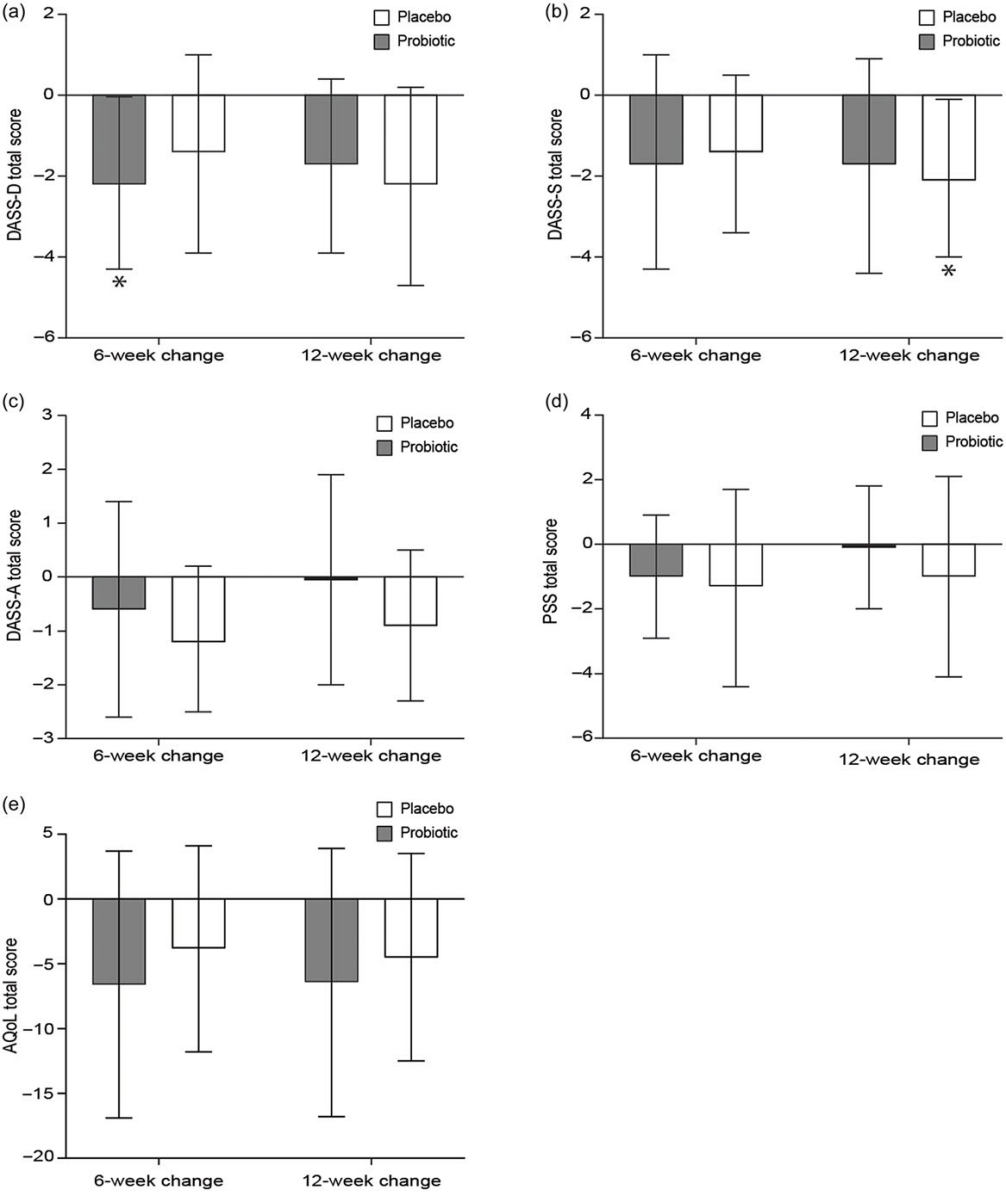
Mean changes (95 % CI) of depression, anxiety, stress and quality of life scores in people diagnosed with subthreshold depression receiving either a probiotic food supplement (*n* 19) or a placebo (*n* 20), from baseline to 6 and 12 weeks of intervention. These changes refer to (a) DASS-D, Depression, Anxiety and Stress Scale-D scores, (b) DASS-S, Depression, Anxiety and Stress Scale-S scores, (c) DASS-A, Depression, Anxiety and Stress Scale-A scores, (d) PSS, Perceived Stress Scale scores and (e) AQoL, Assessment of Quality of Life scores. *P < 0·05, significant within-group change from baseline to follow-up. No significant treatment × time interaction effect was observed at 6 or 12 weeks.

Figures 3 and 4 (online Supplementary Table 1) summarise the within-group changes, the between-group differences and the treatment × time interaction effects in the examined depression, anxiety, stress and quality of life scores. No significant between-group differences or treatment × time interaction effects were observed for these specific outcomes. However, the probiotic group decreased their BDI score by −6·5 (95 % CI –12·3, −0·7) and −7·6 (95 % CI −13·4, −1·8) at 6 and 12 weeks of intervention, respectively. A decrease of −6·6 (95 % CI −12·1, −1·1) was also observed in the placebo group, but only at the 12-week time point. The PHQ score decreased in both groups and more specifically by −4·2 (95 % CI −7·1, −1·3) and −4·1 (95 % CI −7·0, −1·2) in the probiotic group and by −2·5 (95 % CI −4·9, −0·006) and −3·2 (95 % CI −5·7, −0·7) in the placebo group after 6 and 12 weeks of intervention respectively. The HADS Anxiety (HADS-A) score decreased only in the probiotic group by −2·8 (95 % CI −5·2, −0·4) and −2·7 (95 % CI −5·1, −0·3) at 6 and 12 weeks of intervention, respectively. Similarly, the HADS Depression (HADS-D) score decreased in the probiotic group only by −3·0 (95 % CI −5·4, −0·7) and −2·5 (95% CI –4·9, −0·2) at 6 and 12 weeks of intervention, respectively. Furthermore, the DASS Depression (DASS-D) score also decreased in the probiotic group by −2·2 (95 % CI –4·3, −0·04), but only for the first 6 weeks of intervention. On the contrary, the DASS Stress (DASS-S) score decreased only in the placebo group by −2·1 (95 % CI –4·0, −0·1) during the 12-week intervention. No other statistically significant changes or differences were observed within or between groups, respectively.

At the optional 6-week follow-up time point, the probiotic group showed significant decreases in their BDI total score by −7·5 (95 % CI –15·0, −0·005) and in the HADS-D score by −3·6 (95 % CI –6·4, −0·8; *P* = 0·037) compared with the placebo group (online Supplementary Table 2).

### Biochemical outcomes

**Fig. 5.**
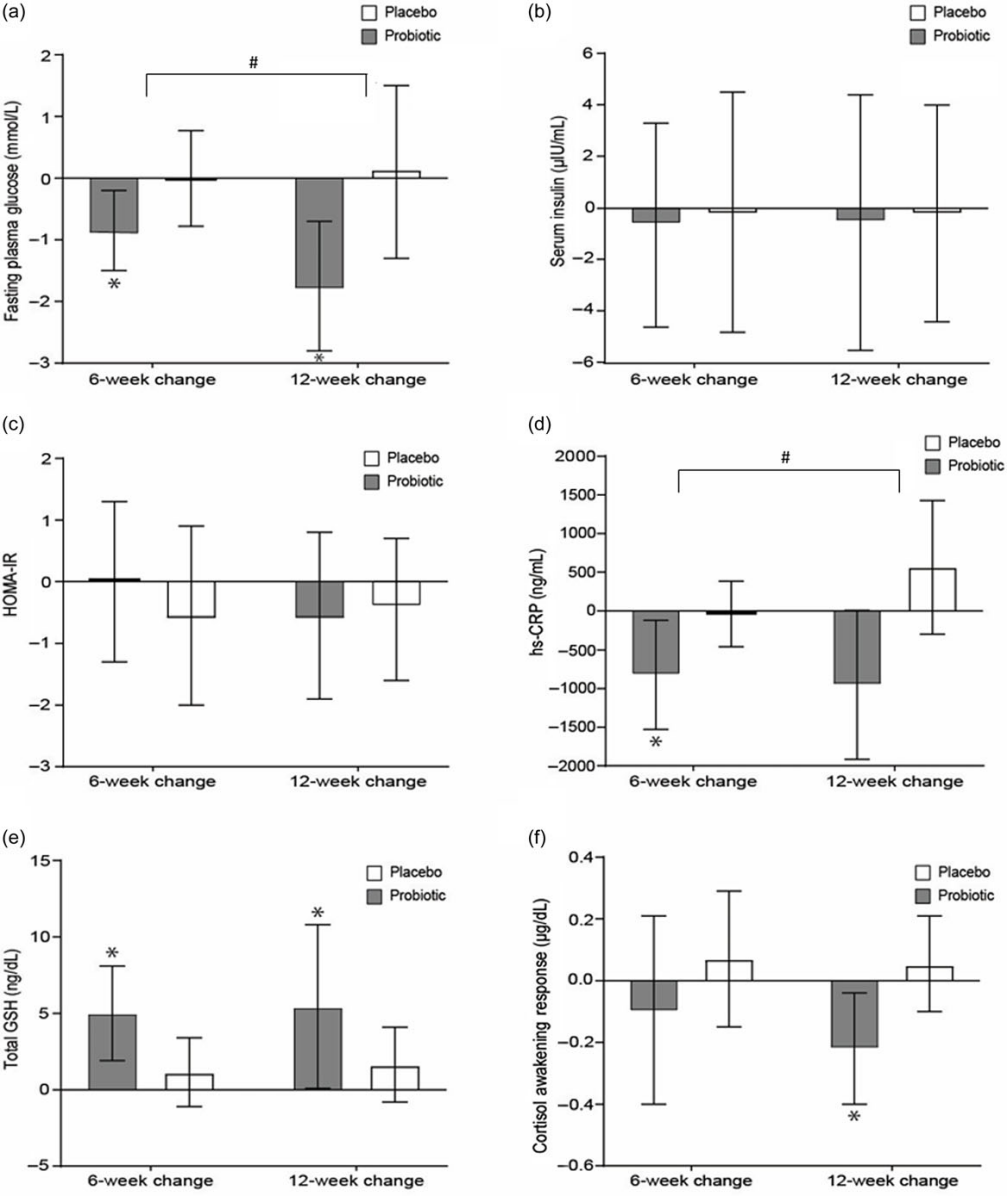
Mean changes (95 % CI) of inflammatory, immune and stress response biomarkers in adults with subthreshold depression receiving either a probiotic food supplement (*n* 19) or a placebo (*n* 20), from baseline to 6 and 12 weeks of intervention. These changes refer to (a) fasting plasma glucose, (b) serum insulin, (c) HOMA-IR, Homeostasis Model Assessment Insulin Resistance, (d) serum hs-CRP, high-sensitivity C-reactive protein, (e) total GSH, glutathione concentrations and (f) saliva CAR, cortisol awakening response. *P < 0·05, significant within-group change from baseline to follow-up. #*P* < 0·05, significant treatment × time interaction effects observed.

Figure 5 (online Supplementary Table 3) presents the within-group changes, the between-group differences and the treatment × time interaction effects in fasting plasma glucose, serum insulin, HOMA-IR (i.e. an index of insulin resistance), serum hs-CRP (i.e. a biomarker of inflammation), total GSH concentrations (i.e. a peptide with antioxidant/protective properties, especially in brain cells) and saliva CAR (i.e. a marker of stress levels). Fasting plasma glucose decreased only in the probiotic group by −0·9 mmol/l (95 % CI –1·5, −0·2) and by −1·8 mmol/l (95 % CI –2·8, −0·7) after 6 and 12 weeks of treatment, respectively. The decrease observed in the probiotic group after 12 weeks of intervention was higher compared with the change observed in the placebo group (–1·8 *v*. 0·1; treatment × time interaction *P* = 0·036). Regarding serum hs-CRP concentrations, they decreased only in the probiotic group by −822·6 ng/ml (95% CI –1528·2; −116·9) after 6 weeks of treatment. Furthermore, the decrease observed in hs-CRP was higher in the probiotic group after 12 weeks of treatment, compared with the relevant increase observed in the placebo group (–954·6 ng/ml *v*. 565·4 ng/ml; treatment × time interaction *P* = 0·047). In addition, the 12-week changes observed in the two treatment groups also led to lower hs-CRP levels in the probiotic compared with the placebo group (7286·2 (sd 1205·8) *v*. 5976·4 (sd 1408·3); *P* = 0·003). Increases in total GSH of 5·0 ng/dl (95% CI 1·9; 8·1) and 5·4 ng/dl (95% CI 0·1; 10·8) were observed only in the probiotic group after 6 and 12 weeks of treatment, respectively. These increases also resulted in a higher mean total serum GSH concentrations at the 6-week (13·8 (sd 5·0) *v*. 8·7 (sd 2·8); *P* = 0·006) and 12-week (14·2 (sd 8·9) *v*. 9·3 (sd 4·7); *P* = 0·049) follow-up time points in the probiotic compared with the placebo group. Within-group changes and between-group differences in the CAR are also presented in Fig. 5. The mean levels of CAR at the end of the 12-week intervention were lower in the probiotic compared with the placebo group (–0·04 (sd 0·17) *v*. 0·16 (sd 0·25); *P* = 0·009), while the decrease of −0·22 μg/dl (95 % CI –0·40, −0·04) in CAR levels from baseline to 12 weeks observed in the probiotic group was different compared with the increase of 0·05 μg/dl (96 % CI –0·10; 0·21) in the placebo group (*P* = 0·038).

## Discussion

This is the first study to examine the effects of a specific multi-strain probiotic supplement on changes related to depression, stress and anxiety scores, as well as blood and saliva biomarkers of glycaemia, inflammation, oxidation status and stress levels, in people with SD. In this double-blind, placebo-controlled, randomised, 12-week trial, the probiotic supplementation decreased depression and anxiety scores at 6 and 12 weeks, effects that were not seen with placebo. These reductions in depression and anxiety scores, which reflect improvements in the relevant symptoms, remained at the 6-week post-intervention follow-up. No changes in perceived stress or quality of life scores occurred. The changes seen in psychometric parameters were supported by physiological biomarkers, finding the probiotic group had lower CAR, reduced hs-CRP and increased total GSH compared with placebo post-intervention. These findings have important clinical relevance given the pressing need for enhanced management of SD, due to patients with SD representing a significant portion (10–35 %) of general practitioners’ caseloads^(46,47)^, and higher missed workdays compared with those without depressive symptoms^(11,46)^. Additionally, the increased likelihood of SD worsening to MDD if left untreated^(10)^ suggest that our findings have potential to lead to a safe and effective prevention option of depression that can be easily implemented to clinical practice.

The effect on mood seen in the current trial in SD are consistent with results from studies in healthy volunteers using the same probiotic formulation. At both 6 and 12 weeks of intervention, the current study found improvements in all measures including depression (measured via BDI, PHQ and HADS-D) and anxiety (measured via HADS-A), with DASS scores also improved at the 6-week time point. Similar data were found in thirty-eight healthy volunteers following a 6-week intervention, whereby Profile of Mood State scores showed lower depression, anger-hostility and fatigue^(32)^. Although BDI scores were unchanged, this might be expected given the population were healthy controls^(32)^. Similarly, the current study did not find differences in the stress (measured via the PSS) or quality of life scores, which may be explained by low levels at baseline which were unable to be lowered further by probiotic intake. The lack of between-group differences noted in the current study is similar to that found in another study of 4-week multi-strain probiotic in twenty healthy participants, where time–group interactions showed reduced self-reported cognitive reactivity to sad mood, although no differences were found between placebo and probiotic groups for depression and anxiety^(26)^. The lack of between-group differences in the current study and those reported previously may represent the milder nature of symptoms in the population studied, with none meeting the clinical cut-off score for more moderate/severe depression. Importantly, and despite the smaller number of participants who completed the 6-week post-intervention follow-up data collection, the improvements observed during the intervention remained in the probiotic group. Overall, the findings are in line with a growing body of evidence showing beneficial effects of psychobiotics on depression symptoms, including meta-analytic data (although these authors concluded no specificity regarding strain, dosage nor treatment duration)^(48)^ and may also indicate longer-term effectiveness.

Previous studies have suggested that inflammation mediates stress-induced depression, including peripheral levels of CRP repeatedly associated with depression^(49)^, and depression or depressive episodes may affect cortisol dysregulation^(50)^. We observed significant improvements in hs-CRP, total GSH, fasting plasma glucose and CAR post-probiotic intervention in adults with sd. It is notewirthy that the significant reduction observed in fasting plasma glucose after the probiotic supplementation resulted to normal plasma glucose concentrations in this group. This is indicative of the effectiveness of the probiotc administered to study participants in ther glycemic control, a finding which to the best of our knowledge has not been previously reported by other trials examining the effectiveness of probiotics on mental health. The findings of the current study also elucidate the biological pathways through which multi-strain probiotics modulate psychosocial health and biomarkers of inflammatory, immune and stress response. Our study aligns with existing literature linking hs-CRP to depression^(49)^ and supports the inflammatory model of depression. Decreased serum hs-CRP has been reported in various patient groups post-probiotic intervention^(45,51)^. GSH improvement suggests potential amelioration of oxidative stress previously linked to anhedonia severity in patients with MDD^(52)^. Future analysis should validate these anti-inflammatory effects through SCFA and inflammatory biomarkers. Unlike some prior studies reporting changes in HOMA-IR or serum insulin^(45)^, we found no such alterations. This may be attributed to our cohort being normoglycaemic, with healthy insulin resistance status, indicating possible condition-specific effects. Improvement in CAR was noted after 12 weeks, corroborating the gradual modification of microbiota composition^(48)^. Previous studies yielded insignificant effects of probiotics on urinary cortisol levels, but our focus on salivary cortisol allows for more sensitive short-term change detection^(53)^.

It is critical to note that the current blood and saliva biomarker findings were not subjected to any ‘placebo-effect’, strengthening the results’ validity. Our findings show a link between biomarkers of inflammation, oxidative stress and depressive symptoms and align with previous studies^(54–56)^.

The reductions in depressive symptoms and anxiety found within the current study offer some hope for potential use as a therapeutic strategy for SD and align with previous interventions with multi-species probiotics showing reduced cognitive reactivity to sad mood in a triple blind, randomised, placebo-controlled study^(26)^. However, the lack of between-group differences in the examined questionnaire scores that reflect depressive symptoms severity observed in the current study when comparing the probiotic and placebo groups means that results should be interpreted cautiously. This may have been due to placebo effects occurring within the placebo group, especially given the self-reported nature of the psychometric results. This would be supported by the non-significant changes observed in the placebo group for the salivary and blood parameters. Studies have suggested a 30 % (range 13–52 %) placebo response rate in studies of major depression^(57,58)^. Despite this lack of between-group differences, the changes seen in the probiotic group are sufficient to consider this probiotic as a potentially effective strategy to improve mood. The probiotic may be considered as an alternative or in combination with other recommended pharmacological therapy, especially given its lack of unwanted side effects, and relative ease of implementation. Patients with low-level symptoms who prefer to rely on their own self-help strategies^(59,60)^ may be the most suitable candidates. However, longer-term studies including cost–benefit analysis will be required prior to addition of such therapy to clinical guidelines.

### Strengths and limitations

There are many strengths of this study which should be considered in its interpretation. First, the gold standard design is used, that is, a double-blinded, placebo-controlled, randomised controlled trial. Second, intention-to-treat analysis was conducted to compensate for missing values and increase statistical power. Third, strict inclusion and exclusion criteria were used, and importantly rather than relying on screening tools, this study included gold standard assessment by a psychologist using the DSM-5 to ensure accurate diagnoses of SD. Furthermore, multiple tools were used to assess the primary end point of depressive symptom severity to ensure increased validity and reduced bias, hence providing a more balanced and comprehensive assessment. Although the use of multiple tools can complicate the interpretation of findings if results are contradictory, we followed our *a priori* criteria and our results showed consistent findings between the BDI and HADS. This is of particular importance considering that the primary end point relied on self-reported data. Fourth, the participant cohort is likely to be representative of the population given the broad advertising strategies used, evidenced by some participants enrolling who had not approached their General Practitioner yet for complaints of their low mood or mild depression. Finally, adverse events and compliance to the intervention were recorded for the majority of study participants. Unfortunately, compliance could not be assessed for the entire study population, meaning those who did not report compliance may have been less compliant and consequently created a bias. Although our saliva sampling protocol adhered to most of the expert consortium guidelines^(43)^, we do acknowledge that objective measures of waking, time-capped tubes and additional sampling points may have provided additional rigour. Other study limitations include that the length of the intervention (3 months) does not allow for understanding of the probiotic’s role in the longer term, although this was beyond the scope of this study. Furthermore, while the total sample size was only one participant lower than the estimated requirement (i.e. 39 instead of 40), the non-significant between-group differences observed in all mood-related outcomes possibly indicate that the actual effect size was not providing enough statistical power. Additionally, although common in studies of this nature^(26)^, there were more females enrolled compared with males; hence, this may limit generalisability of the findings. Finally, the higher education level in the probiotic group may have influenced findings, although it would not be anticipated to have affected any physiological markers.

Given our findings, several avenues for future research and clinical implications emerge. To build upon the beneficial effects of multi-strain probiotics on psychosocial health, subsequent studies should examine faecal microbiota composition differences in patients with MDD compared with healthy controls^(61)^. Future investigations could probe into neurotransmitters or their precursors as additional mechanistic pathways for understanding the influence of probiotics on depressive symptoms. Expanding the focus to other emotional processes, sleep quality and fatigue measures could provide a holistic understanding, especially given the known interplay between sleep quality and mood. Longer-term intervention or follow-up studies are warranted, including understanding the sustainability of observed improvements and potential recurrence of depressive symptoms upon cessation of probiotic intake. Finally, utilisation of proteomic, socio-demographic and clinical data in predictive models could enhance personalised treatment approaches for SD^(62)^.

In summary, a significant sustainable improvement in depressive symptoms and anxiety was observed within the probiotic group, both during the 12-week intervention and the 6-week post-intervention follow-up. The 12-week improvements in depressive symptoms and anxiety were supported by observed improvements in biomarkers, suggesting that probiotics may support psychological well-being in adults experiencing SD and may exert their beneficial effects via cortisol regulation, inflammatory modulation and oxidative stress reduction. Although the within-group changes observed in the probiotic group were supported by favourable improved inflammatory, antioxidant status and stress biomarkers compared with the placebo, they are only exploratory and should be interpreted with caution. Future research is required to assess longer-term effects and understand changes within the intestinal microbiota and to clarify how their metabolites facilitate emotional regulation and psychological well-being.

## Supporting information

Moschonis et al. supplementary material 1 Moschonis et al. supplementary material

Moschonis et al. supplementary material 2 Moschonis et al. supplementary material

## Supplementary material

For supplementary material/s referred to in this article, please visit https://doi.org/10.1017/S0007114524001703
