# Supplementary material for: Evaluation of a probiotic blend on psychosocial health and biomarkers of inflammatory, immune and stress response in adults with subthreshold depression: a double-blind, randomised, placebo-controlled trial
Source: Br J Nutr. 2024 Oct 29;135(5):481–95. doi: 10.1017/S0007114524001703 (PMC13161528; doi:10.1017/S0007114524001703)
Supplement: Moschonis et al. supplementary material 1 — Moschonis et al. supplementary material [file S0007114524001703sup001.docx]

**Supplementary Table 1.** Mean changes in the severity of depressive symptoms, anxiety, stress, and quality of life in people diagnosed with subthreshold depression receiving either a probiotic food supplement (n=19) or a placebo (n=20), from baseline to 6 and 12 weeks of intervention.

|  | **Baseline** | **6-week**  **Follow-up** | **6-Week**  **Change** | **12-Week**  **Follow-up** | **12-Week**  **Change** |
| --- | --- | --- | --- | --- | --- |
|  | Mean (SD) | Mean (SD) | Mean (95% CI) | Mean (SD) | Mean (95% CI) |
| **BDI total score** |  |  |  |  |  |
| Placebo | 23.8 (9.7) | 19.0 (9.1) | -4.8 (-10.3; 0.6) | 17.2 (6.8) | **-6.6 (-12.1; -1.1)** |
| Probiotic | 23.4 (9.3) | 16.9 (8.2) | **-6.5 (-12.3; -0.7)** | 15.8 (9.2) | **-7.6 (-13.4; -1.8)** |
| *P-*value^†^ | 0.889 | 0.450 | 0.451^*^ | 0.592 | 0.747^*^ |
| **PHQ total score** |  |  |  |  |  |
| Placebo | 10.3 (4.6) | 7.9 (3.8) | **-2.5 (-4.9; -0.006)** | 7.1 (3.0) | **-3.2 (-5.7; -0.7)** |
| Probiotic | 11.9 (4.6) | 7.6 (4.3) | **-4.2 (-7.1; -1.3)** | 7.8 (4.4) | **-4.1 (-7.0; -1.2)** |
| *P-*value^†^ | 0.299 | 0.856 | 0.235^*^ | 0.602 | 0.583^*^ |
| **HADS-A total score** |  |  |  |  |  |
| Placebo | **7.7 (3.1)** | 6.9 (3.2) | -0.8 (-2.9; 1.2) | 6.7 (3.5) | -1.0 (-3.0; 1.0) |
| Probiotic | **11.4 (3.6)** | 8.6 (3.4) | **-2.8 (-5.2; -0.4)** | 8.7 (4.0) | **-2.7 (-5.1; -0.3)** |
| *P-*value^†^ | **0.001** | 0.106 | 0.059^*^ | 0.094 | 0.231^*^ |
| **HADS-D total score** |  |  |  |  |  |
| Placebo | 7.4 (2.8) | 5.8 (3.5) | -1.6 (-3.6; 0.3) | 6.6 (2.9) | -0.8 (-2.7; 1.2) |
| Probiotic | 8.6 (3.9) | 5.6 (3.0) | **-3.0 (-5.4; -0.7)** | 6.1 (3.8) | **-2.5 (-4.9; -0.2)** |
| *P-*value^†^ | 0.268 | 0.854 | 0.166^*^ | 0.636 | 0.096^*^ |
| **DASS-D total score** |  |  |  |  |  |
| Placebo | 7.4 (4.4) | 6.0 (4.0) | -1.4 (-3.9; 1.0) | 5.2 (2.9) | -2.2 (-4.7; 0.2) |
| Probiotic | 7.6 (3.8) | 5.4 (2.5) | **-2.2 (-4.3; -0.04)** | 5.9 (3.5) | -1.7 (-3.9; 0.4) |
| *P-*value^†^ | 0.861 | 0.624 | 0.451^*^ | 0.489 | 0.706^*^ |
| **DASS-A total score** |  |  |  |  |  |
| Placebo | 3.8 (2.4) | 2.7 (1.9) | -1.2 (-2.5; 0.2) | 2.9 (2.2) | -0.9 (-2.3; 0.5) |
| Probiotic | 4.4 (3.4) | 3.8 (2.5) | -0.6 (-2.6; 1.4) | 4.4 (3.2) | -0.01 (-2.0; 1.9) |
| *P-*value^†^ | 0.548 | 0.115 | 0.563^*^ | 0.097 | 0.423^*^ |
| **DASS-S total score** |  |  |  |  |  |
| Placebo | 7.6 (3.2) | 6.2 (2.8) | -1.4 (-3.4; 0.5) | 5.5 (3.1) | **-2.1 (-4.0; -0.1)** |
| Probiotic | 8.6 (4.2) | 6.9 (4.2) | -1.7 (-4.3; 1.0) | 6.8 (3.9) | -1.7 (-4.4; 0.9) |
| *P-*value^†^ | 0.416 | 0.518 | 0.841^*^ | 0.255 | 0.804^*^ |
| **PSS total score** |  |  |  |  |  |
| Placebo | 20.5 (4.4) | 19.2 (5.9) | -1.3 (-4.4; 1.7) | 19.5 (4.1) | -1.0 (-4.1; 2.1) |
| Probiotic | 21.4 (3.7) | 20.4 (2.5) | -1.0 (-2.9; 0.9) | 21.3 (2.4) | -0.06 (-2.0; 1.8) |
| *P-*value^†^ | 0.532 | 0.416 | 0.808^*^ | 0.118 | 0.485^*^ |
| **AQoL total score** |  |  |  |  |  |
| Placebo | 81.4 (12.0) | 77.6 (14.0) | -3.8 (-11.8; 4.1) | 76.9 (11.7) | -4.5 (-12.5; 3.5) |
| Probiotic | 84.2 (16.3) | 77.6 (13.7) | -6.6 (-16.9; 3.7) | 77.8 (17.4) | -6.4 (-16.8; 3.9) |
| *P-*value^†^ | 0.542 | 0.986 | 0.499^*^ | 0.859 | 0.661^*^ |

^*^: Treatment x Time interaction effect; ^†^: Between-groups’ differences in mean values at baseline, 6 and 12 weeks, as well as in 6- and 12-week changes from baseline (Treatment effect). SD, standard deviation; CI, confidence interval; BDI, Beck Depression Inventory; PHQ, Patient Health Questionnaire; HADS-A, Hospital Anxiety and Depression Scale-Anxiety; HADS-D, Hospital Anxiety and Depression Scale-Depression; DASS-D, Depression, Anxiety and Stress Scale-Depression; DASS-A, Depression Anxiety and Stress Scale-Anxiety; DASS-S, Depression, Anxiety and Stress Scale-Stress; PSS-Perceived Stress Scale; AQoL, Assessment of Quality of Life. Results in bold indicate statistical significance (p<0.05). **Note:** Decreases in the scores are indicative of improvements in the relevant depressive symptoms, anxiety, stress, and quality of life that each score measures.

**Supplementary Table 3.** Changes in biochemical markers of glycaemic profile, inflammation, oxidative stress, and saliva cortisol levels in people diagnosed with subthreshold depression receiving either a probiotic food supplement (n=19 for blood biomarkers and n=18 for saliva cortisol) or a placebo (n=19 for blood biomarkers and n=20 for saliva cortisol), from baseline to 6 and 12 weeks of intervention.

|  | **Baseline** | **6-week**  **Follow-up** | **6-Week**  **Change** | **12-Week**  **Follow-up** | **12-Week**  **Change** |
| --- | --- | --- | --- | --- | --- |
|  | Mean (SD) | Mean (SD) | Mean (95% CI) | Mean (SD) | Mean (95% CI) |
| **Fasting Plasma Glucose (mmol/L)** |  |  |  |  |  |
| Placebo | 6.3 (4.3) | 6.3 (3.9) | -0.01 (-0.78; 0.77) | 6.4 (4.8) | 0.1 (-1.3; 1.5) |
| Probiotic | 6.2 (3.3) | 5.3 (3.2) | **-0.9 (-1.5; -0.2)** | 4.4 (2.9) | **-1.8 (-2.8; -0.7)** |
| *P-*value^†^ | 0.918 | 0.419 | 0.070* | 0.347 | **0.036*** |
| **Serum Insulin (μIU/mL)** |  |  |  |  |  |
| Placebo | 9.7 (8.0) | 9.5 (6.0) | -0.2 (-4.8; 4.5) | 9.5 (6.8) | -0.2 (-4.4; 4.0) |
| Probiotic | 9.1 (5.2) | 8.5 (6.2) | -0.6 (-4.6; 3.3) | 8.6 (7.6) | -0.5 (-5.5; 4.4) |
| *P-*value^†^ | 0.821 | 0.785 | 0.715^*^ | 0.542 | 0.821^*^ |
| **HOMA-IR** |  |  |  |  |  |
| Placebo | 3.2 (3.6) | 2.6 (2.0) | -0.6 (-2.0; 0.9) | 2.7 (3.0) | -0.4 (-1.6; 0.7) |
| Probiotic | 2.6 (2.1) | 2.6 (3.4) | 0.01 (-1.3; 1.3) | 2.0 (2.6) | -0.6 (-1.9; 0.8) |
| *P-*value^†^ | 0.477 | 0.905 | 0.585* | 0.744 | 0.854* |
| **hs-CRP (ng/mL)** |  |  |  |  |  |
| Placebo | 6720.8 (1190.7) | 6682.5 (1600.5) | -38.3 (-459.8; 383.2) | 7286.2 (1205.8) | 565.4 (-297.4; 1428.3) |
| Probiotic | 6931.0 (1517.2) | 6108.4 (1540.0) | **-822.6 (-1528.2; -116.9)** | 5976.4 (1408.3) | -954.6 (-1914.9; 5.69) |
| *P-*value^†^ | 0.850 | 0.157 | 0.054^*^ | **0.003** | **0.047^*^** |
| **Total GSH (ng/dL)** |  |  |  |  |  |
| Placebo | 7.7 (3.8) | 8.7 (2.8) | 1.1 (-1.1; 3.4) | 9.3 (4.7) | 1.6 (-0.8; 4.1) |
| Probiotic | 8.8 (4.1) | 13.8 (5.0) | **5.0 (1.9; 8.1)** | 14.2 (8.9) | **5.4 (0.1; 10.8)** |
| *P-*value^†^ | 0.719 | **0.006** | **0.046^*^** | **0.049** | 0.198^*^ |
| **Cortisol Awakening Response (μg/dL)** |  |  |  |  |  |
| Placebo | 0.11 (0.34) | 0.18 (0.34) | 0.07 (-0.15; 0.29) | **0.16 (0.25)** | 0.05 (-0.10; 0.21) |
| Probiotic | 0.18 (0.35) | 0.08 (0.44) | -0.10 (-0.40; 0.21) | **-0.04 (0.17)** | **-0.22 (-0.40; -0.04)** |
| *P-*value^†^ | 0.460 | 0.137 | 0.106^*^ | **0.009** | **0.038^*^** |

^*^: Treatment x Time interaction effect; ^†^: Between-groups’ differences in mean values at baseline, 6 and 12 weeks, as well as in 6- and 12-week changes from baseline (Treatment effect). Adjustments were made for study participants’ educational level, changes in dietary energy intake from baseline to 6 weeks and from baseline to 12 weeks, change in dietary protein intake from baseline to 12 weeks and mean dietary fibre intake at 6 weeks. SD, standard deviation; CI, confidence interval; HOMA-IR, Homeostatic Model Assessment for Insulin Resistance; hs-CRP**:** High sensitivity C-Reactive Protein; GSH: Total Glutathione. Results in bold indicate statistical significance (p<0.05).
