# Supplementary material for: Evaluation of a probiotic blend on psychosocial health and biomarkers of inflammatory, immune and stress response in adults with subthreshold depression: a double-blind, randomised, placebo-controlled trial
Source: Br J Nutr. 2024 Oct 29;135(5):481–95. doi: 10.1017/S0007114524001703 (PMC13161528; doi:10.1017/S0007114524001703)
Supplement: Moschonis et al. supplementary material 2 — Moschonis et al. supplementary material [file S0007114524001703sup002.docx]

**Supplementary Table 2.** Changes in the severity of depressive symptoms, other measures of psychosocial health and quality of life in people diagnosed with subthreshold depression receiving either a probiotic food supplement (n=10) or a placebo (n=9), from baseline to the 18-week follow-up (6 weeks post-intervention).

|  | **Baseline** | **18-Week Follow-up**  **(6 weeks post-intervention)** | **18-Week**  **Change** |
| --- | --- | --- | --- |
|  | Mean (SD) | Mean (SD) | Mean (95% CI) |
| **BDI total score** |  |  |  |
| Placebo | 20.9 (7.5) | 18.8 (10.8) | -2.1 (-8.9; 4.7) |
| Probiotic | 22.8 (10.1) | 15.3 (12.4) | **-7.5 (-15.0; -0.005)** |
| *P-*value^†^ | 0.650 | 0.525 | 0.245^*^ |
| **PHQ total score** |  |  |  |
| Placebo | 9.3 (3.7) | 8.3 (4.2) | -1.0 (-5.4; 3.4) |
| Probiotic | 12.3 (5.1) | 10.0 (7.4) | -2.3 (-6.1; 1.4) |
| *P-*value^†^ | 0.171 | 0.563 | 0.611^*^ |
| **HADS-A total score** |  |  |  |
| Placebo | 6.3 (2.1) | 8.3 (4.4) | 2.0 (-1.0; 5.0) |
| Probiotic | 10.5 (3.2) | 8.1 (5.3) | -2.4 (-5.6; 0.8) |
| *P-*value^†^ | **0.004** | 0.919 | **0.037^*^** |
| **HADS-D total score** |  |  |  |
| Placebo | 7.7 (2.3) | 7.7 (4.0) | 0.0 (-3.0; 3.0) |
| Probiotic | 8.8 (4.1) | 5.2 (4.9) | **-3.6 (-6.4; -0.8)** |
| *P-*value^†^ | 0.479 | 0.250 | 0.062^*^ |
| **HADS total score** |  |  |  |
| Placebo | **14.0 (2.7)** | 16.0 (6.7) | 2.0 (-3.5; 7.5) |
| Probiotic | **19.3 (7.0)** | 13.3 (9.6) | **-6.0 (-11.7 -0.3)** |
| *P-*value^†^ | **0.048** | 0.491 | **0.035^*^** |
| **DASS-D total score** |  |  |  |
| Placebo | 7.7 (4.2) | 7.4 (5.1) | -0.3 (-2.7; 2.3) |
| Probiotic | 7.9 (4.5) | 6.0 (5.4) | -1.9 (-6.3; 2.5) |
| *P-*value^†^ | 0.909 | 0.558 | 0.474^*^ |
| **DASS-A total score** |  |  |  |
| Placebo | 2.7 (2.2) | 2.8 (2.0) | 0.1 (-0.9; 1.1) |
| Probiotic | 3.4 (3.4) | 4.0 (4.8) | 0.6 (-1.2; 2.4) |
| *P-*value^†^ | 0.591 | 0.486 | 0.610^*^ |
| **DASS-S total score** |  |  |  |
| Placebo | 6.8 (2.3) | 7.0 (2.9) | 0.2 (-1.6; 2.0) |
| Probiotic | 9.1 (4.3) | 7.3 (6.4) | -1.8 (-5.5; 1.9) |
| *P-*value^†^ | 0.167 | 0.899 | 0.294^*^ |
| **PSS total score** |  |  |  |
| Placebo | 19.2 (4.8) | 20.6 (3.0) | 1.4 (-1.5; 4.1) |
| Probiotic | 22.2 (3.5) | 21.4 (3.3) | -1.2 (-5.5; 3.5) |
| *P-*value^†^ | 0.138 | 0.560 | 0.324^*^ |
| **AQoL total score** |  |  |  |
| Placebo | 82.7 (12.2) | 76.2 (16.8) | -6.4 (-16.2; 3.3) |
| Probiotic | 87.5 (17.7) | 76.6 (24.9) | -10.9 (-27.3; 5.5) |
| *P-*value^†^ | 0.502 | 0.970 | 0.614^*^ |

^*^: Treatment x Time interaction effect; ^†^: Between-groups’ differences in mean values at baseline, 18 weeks, as well as in 18-week changes from baseline (Treatment effect). **Note:** As participation at this post-intervention time-point was optional, data was collected from 19 participants (n=10 probiotic and n=9 placebo) out of the total n=39. Considering that the sample size did not allow us to conduct an Intention-to-treat analysis (this requires at least 80% of the original sample), the results presented in come from the per-protocol analysis on the 19 participants.
